# Mechanical and Acoustic Emission Behavior of Gangue Concrete under Uniaxial Compression

**DOI:** 10.3390/ma12203318

**Published:** 2019-10-11

**Authors:** Meng Xiao, Feng Ju, Pai Ning, Kaiyuan Li

**Affiliations:** State Key Laboratory for Geomechanics & Deep Underground Engineering, China University of Mining and Technology, Xuzhou 221116, China; m.xiao@cumt.edu.cn (M.X.); 17851146436@163.com (P.N.); TS18030022A31@cumt.edu.cn (K.L.)

**Keywords:** concrete, coal gangue, acoustic emission, comparison, environment

## Abstract

The application of gangue concrete can be an effective method to solve the massive gangue heap and shortage of raw materials of concrete by replacing the gravel and river sand with crushed gangue. An Acoustic emission (AE) is one of the non-destructive testing methods that can be used for damage detection of the gangue concrete structure. However, there are obvious mechanics differences between gangue and gravel/river sand, so the previous analysis methods of AE signal for concrete structure detection, mainly applied to ordinary concrete, are not suitable for gangue concrete. Based on this, the physical and mechanical characteristics of coal gangue were studied, and the uniaxial compressive test, along with AE monitoring of gangue concrete, was conducted in this paper. The differences in AE behavior between gangue concrete and ordinary concrete were also analyzed. The mechanical test result shows that the compressive strength of gangue concrete can reach 35–40 MPa. Comparing with ordinary concrete, gangue concrete has larger initial porosity and abrupt rupture. Additionally, the accumulative energy growth rate of gangue concrete has two peak values before the peak load, while ordinary concrete only has one. This difference can be used to forecast damage of gangue concrete structure by AE technology. This paper shows the possibility of making concrete by coal gangue, and the possibility of identifying its damage degree with the use of acoustic emission technology.

## 1. Introduction

Concrete, the most widely used building material at present, is cementing composites that use gravels and river sand as aggregate [1]. Concrete’s raw materials, which include river sand and gravel, are mainly excavated from the riverbed and its surrounding area. However, the exploitation of river sand and gravels has caused serious damage to the river’s ecological environment [2,3,4,5]. For these reasons, most governments have determined to restrict the related exploitation engineering, so there is a shortage of concrete materials and its price has risen [6]. On the other hand, gangue is a kind of solid waste produced in coal mining and has a large quantity [7]. According to some statistics, the amount of gangue in China has reached 5 billion tons, and is still increasing by 200 million tons a year [8]. Massive gangue waste has caused a series of pollution problems, which seriously hinder the development of society and the economy [9]. Therefore, new disposal methods of gangue waste are urgently needed. The authors found that it can be an effective method to solve the massive gangue waste and shortage of raw materials of concrete by replacing the gravel and river sand by crushed gangue. This building material can be used for shotcrete support of mine tunnel.

Acoustic emission (AE) is a non-destructive testing method and has some advantages, such as real-time monitoring, reliability, and high sensitivity. In recent years, AE technology has become a frequently-used method to damage detection of concrete structures. It has been more than 30 years since some scientists began conducting the research into AE technology, which acquired many of achievements [10]. At first, many researchers focused on microcracks development laws of concrete though the AE method, and analyzed the macro rupture of concrete by meso-mechanics [11,12,13]. Some scientists established the relationship between AE parameters and damage variable, and quantify the damage level of concrete by AE technology [10,14,15]. Colombo et al. [16] introduced “b-value” from seismology, and quantify the damage to reinforced concrete by this parameter. On his basis, other scientists proposed the method of predicting the crack initial time and describing the change in the process from critical state to ultimate rupture by “b-value” [17,18]. Besides, the AE testing method is often used to evaluate the corrosion of the steel bars embedded in reinforced concrete structures [19,20,21,22]. Recent works mostly related to the classification of concrete microcracks, and predict the critical state of concrete by AE signal analysis [23,24,25,26].

As mentioned above, AE technology has become one of the most widely used non-destructive testing methods in the field of concrete engineering safety detection. AE signal behavior of concrete under different stress states is important to evaluate the damage level of the concrete structure. However, the previous analysis methods of AE signals about concrete structure detection can not apply to gangue concrete, as a result of obvious mechanical differences between gangue and gravel/river sand. Thus, it is important to investigate the AE behavior of gangue concrete. Base on this, the physical and mechanical characteristics of coal gangue was studied, the gangue concrete with higher strength was proposed in this paper, axial compressive tests along with AE detection of gangue concrete were conducted, and the AE signals in concrete damage processes were obtained in this paper. Additionally, the differences in AE behavior between gangue concrete and ordinary concrete were analyzed, and the variation laws of AE parameters in each stage of gangue concrete damage were obtained. This paper aims to study the change law of AE parameters with the increasing of external loading so as to apply AE non-destructive testing technology to gangue concrete in the future.

## 2. Experimental Materials and Methods

### 2.1. Materials

**Figure 1:**
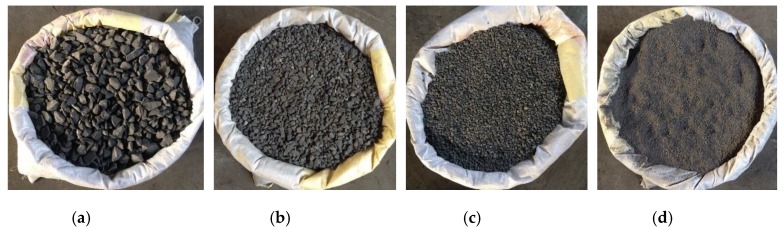
Crushed gangue. (**a**) 10–15 mm; (**b**) 5–10 mm; (**c**) 2.5–5 mm; (**d**) 0–2.5 mm.

**Figure 2:**
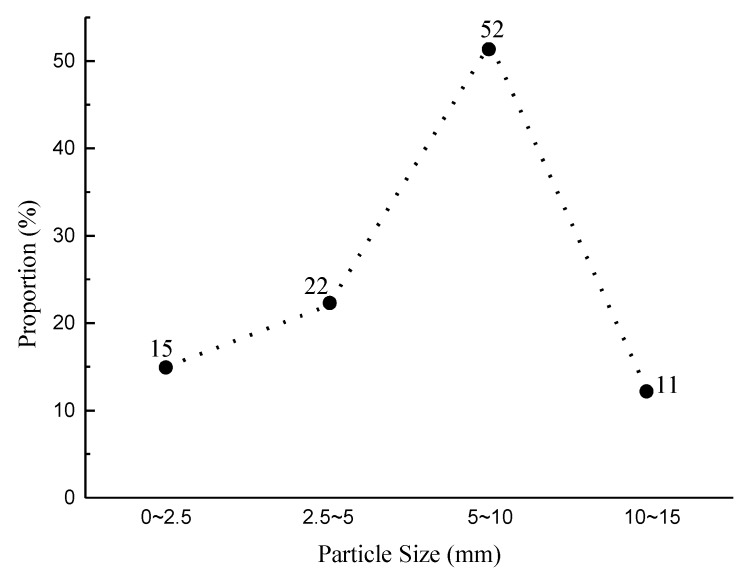
The proportion of each gangue particle size group.

**Figure 3:**
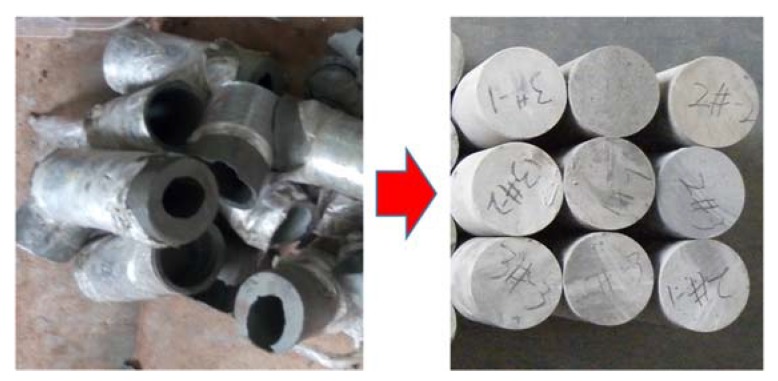
The coal gangue specimens for compressive test.

The gangue used in this study comes from Kailuan (group) Co. LTD, which is a large coal enterprise in Tangshan City, China. Most of the gangue was excavated from dirt bands in the coal seam. The gangue was crushed by a small jaw crusher, and sieved by square hole sieve. And the crushed gangue was divided into four groups by their size, which includes 0–2.5 mm, 2.5–5 mm, 5–10 mm, and 10–15 mm (Figure 1). The proportion of each particle size group was measured as shown in Figure 2, which indicates that the third group (5–10 mm) has the largest proportion, while the fourth group (10–15 mm) has the smallest proportion. The particle size gravel used to prepare ordinary concrete was 5–16 mm. In order to evaluate the strength of coal gangue material, the cylindrical specimens (φ50 mm × 100 mm) were drilled from the coal gangue block (Figure 3).

### 2.2. Mix Proportion

Two types of concrete were chosen in this test, and the detailed parameters are listed in Table 1. These grading types of gangue concrete were demonstrated that have higher strength in the authors’ earlier research. The ordinary concrete’s grading was determined by the Reference [27], and the uniaxial compressive strength of ordinary concrete with this mix proportion is supposed to reach 40–45 MPa. The medium size river sand and P-0 42.5 Portland cement were also used in this test.

In order to obtain the standard strength of concrete, the specimen preparation refers to a national standard [28]. The cube specimens (150 mm × 150 mm × 150 mm) are used in this test.

At first, the crushed gangue was screened and classified as four groups, which have particle sizes of 0–2.5 mm, 2.5–5 mm, 5–10 mm, and 10–15 mm, respectively. According to the parameters in Table 2 and Table 3, gangue (or gravel), sand, cement, and water were weighted and then stirred for 3 min in a small concrete mixer. Engine oil was brushed inside of the mold, and then the mixed concrete was poured into the molds and compacted. After that, the molds were placed on the vibration table for vibration until the bleeding appears on the concrete surface. Finally, these molds were detached after 48 h, and the curing period for these concrete specimens is 28 days in standard curing room [28].

### 2.3. Methods

#### 2.3.1. AE System

**Figure 4:**
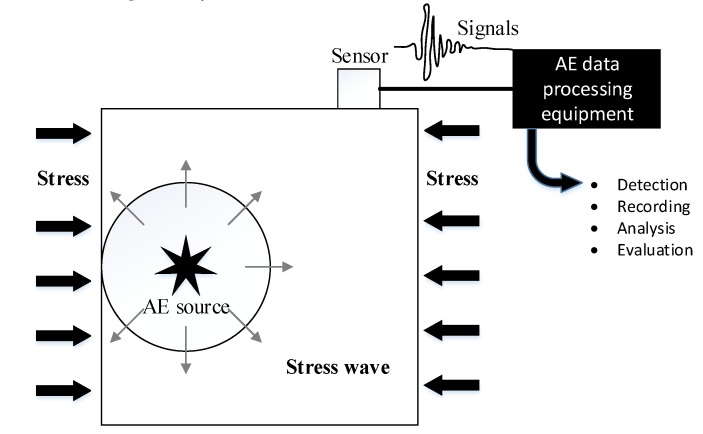
The detection principal of AE system.

Figure 4 indicates the principle of the AE system. When the concrete specimen is under high stress, stress waves induced by microcracks initialize and propagate, spreading from AE sources to the specimen’s surface. These stress waves can be collected by the sensor mounted in the concrete surface and processed by AE processors. Some specific characteristic parameters can be calculated and used for concrete damage analysis.

**Figure 5:**
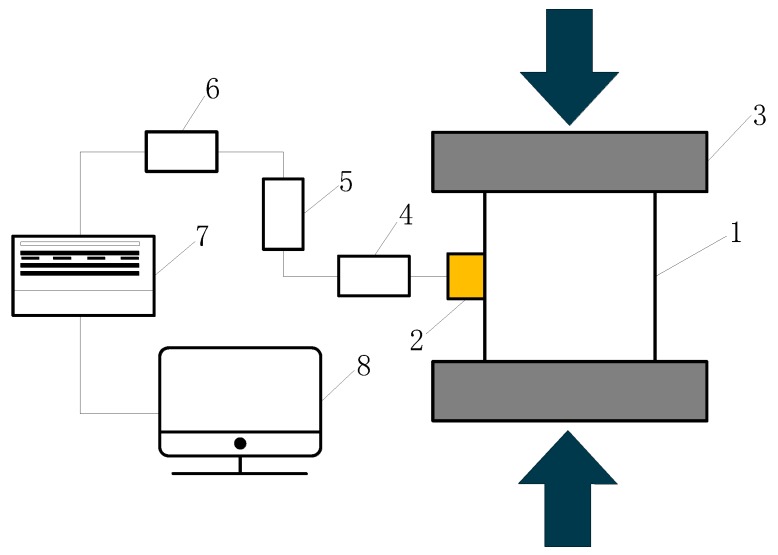
Schematic diagram of the AE measurement: 1. Concrete specimen; 2. AE sensor; 3. Loading platens; 4. AE per-amplifiers; 5. Filters of AE signals; 6. Analog-to-digital convert; 7. Signal acquisition unit; 8. Data analyzer.

In this test, the SEMOS V1.0 seismic-electric-magnetic coupling effects observation system (Anhui Wantai Geophysical Technology Co., Ltd, Hefei City, China) was used (Figure 5). This system is mainly applied for synchronous observation of real-time signals of AE, self-potential, and electromagnetic radiation throughout the deformation and the failure process of standard rock specimens under uniaxial compression or hydrofracture conditions, which is convenient for analysis of evolutionary characteristics of the seismic wavefield, electric field, and magnetic field in rock specimens. It can collect signals of 12 AE channels, 32 electric channels, and 6 transient electromagnetic channels simultaneously. This system has high sampling accuracy, 1.25 MHz real-time sampling frequency, 50 kHz maximum response frequency, 24-bit A/D conversion, dynamic range ≥ 258 dB, and 10^0^–10^6^ time-varying gain [29]. Limited by the experimental purpose, only part of AE monitoring was used in this study.

#### 2.3.2. Loading Machine

**Figure 6:**
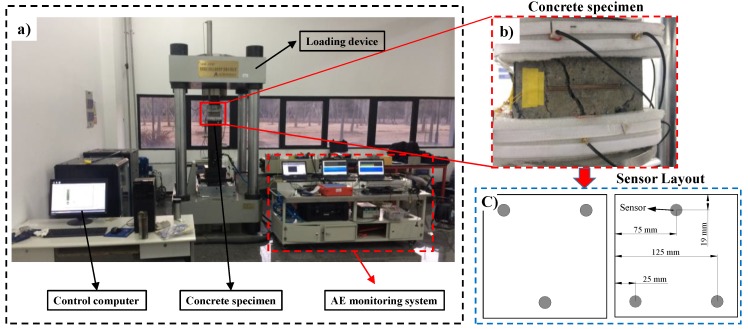
The test equipment layout. (**a**) General arrangement of equipment; (**b**) Concrete specimen; (**c**) AE sensor arrangement.

A hydraulic servo loading device (TAWZ-2000/50, Chaoyang Test Instrument Co., Ltd, Changchun City, China) was used in this test. The maximum axial compressive force can reach 2000 kN, and the maximum piston stroke is 150 mm. The test equipment layout is shown in Figure 6a.

#### 2.3.3. Uniaxial Compressive Test with AE Monitoring

Acoustic emission measurement was conducted during the uniaxial compressive test of gangue concrete. The AE monitoring system is illustrated in Figure 6. Before the test, the rough surface of loading was polished and all dust was removed. The specimens were measured to obtain their precise size. The wideband sensors were attached to the surface of gangue concrete (Figure 6b). The sensors were distributed as shown in Figure 6c. And specially-made grease was used as acoustic couplant to obtain the accurate acoustic signal. After that, the AE data transmitted was verified. AE signals were amplified by 68 dB, and the threshold level was also set to 68 dB. The specimen’s size and type of material were inputted to computer. The strain was calculated automatically by the computer. Preload before the normal test was performed to detect whether the whole system was in good condition. The load control method was applied in the uniaxial compressive test, in which the loading rate was set to 680 N/s. The loading pressure keeps increase until the specimen break.

## 3. Physical and Mechanical Properties of Coal Gangue

### 3.1. Physical Properties of Coal Gangue

**Figure 7:**
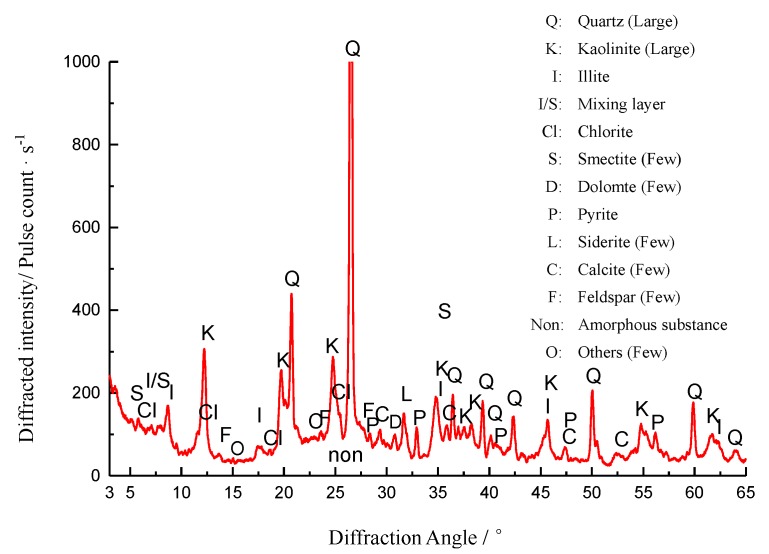
X-Ray diffraction pattern of Gangue.

The mineral composition of gangue was tested by an X-ray diffraction instrument (D/Max-3B, made by Rigaku Co., Tokyo, Japan). The step scan, one of the scan methods of quantitative analysis, was used in this test. The scanning speed and sample interval were set to be 0.25°/min and 0.01°, respectively. The test results are shown in Table 2 and Figure 7. The proportion of quartz and kaolinite is largest, which following with feldspar, illite, illite-smectite mixing layer, and the proportion of chlorite, siderite is very little. The high proportion of quartz and kaolinite shows that coal gangue is mostly shale and sandstone. This is quite different from gravel that mostly limestone.

The chemical component of gangue is shown as Table 3. It indicates that, in coal gangue, SiO_2_ has the highest content, and the higher content of SiO_2_ is also often observed in the gravel. The high content of SiO_2_ shows that the coal gangue can be used as the concrete aggregate. However, the gangue contains few easily weathered and hydrolyzed chemical components, such as Al and CaO.

**Figure 8:**
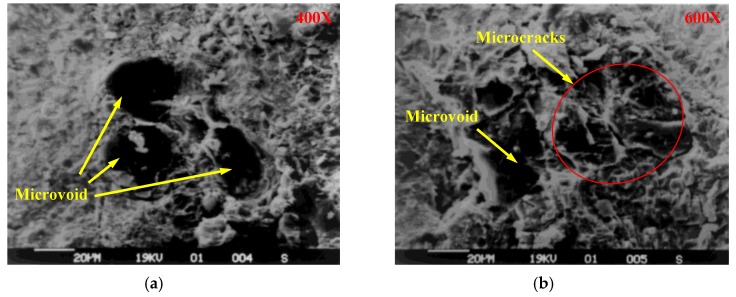
Surface morphology of crushed coal gangue. (**a**) 400× distinguishability; (**b**) 600× distinguishability.

It can be seen by electron microscope scanning (Figure 8) that a small number of microvoids and microcracks exist in the crushed coal gangue. When a high external force is loaded on gangue concrete, the coal gangue may rupture because of the existence of these micro-defects.

### 3.2. Compressive Strength

**Figure 9:**
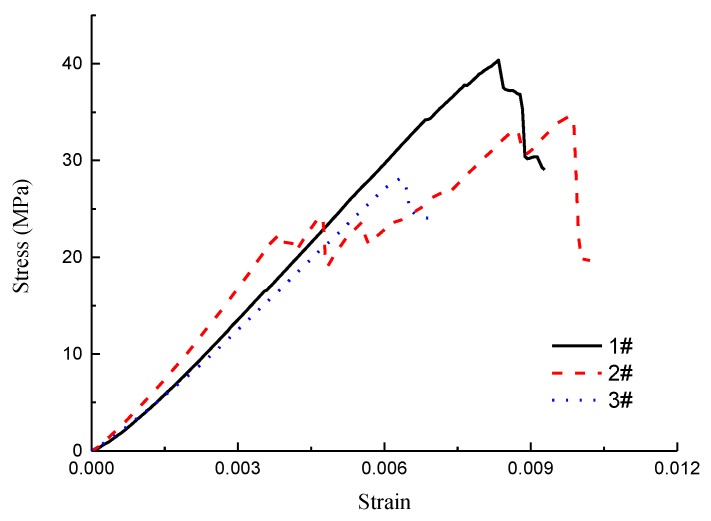
Stress-strain curve of coal gangue specimen.

In order to evaluate the strength of coal gangue, uniaxial compressive experiments were carried out with prepared cylindrical coal gangue specimens. The result (Figure 9) shows that the strengths of the three specimens were 28, 34 and 40 MPa, respectively. The strength of gangue concrete is smaller than the gravel used in ordinary concrete, which is usually higher than 60 MPa.

## 4. Result and Discussion

### 4.1. Mechanics Property of Gangue Concrete

**Figure 10:**
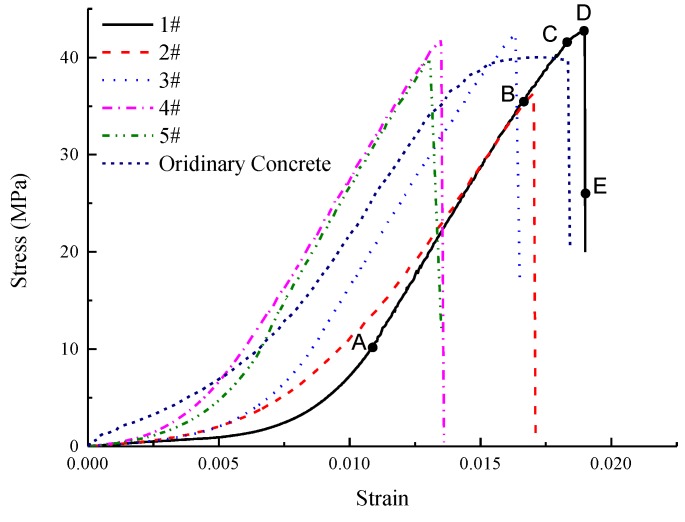
Stress-strain curve of gangue concrete.

Figure 10 shows the stress-strain curves of gangue concrete and ordinary concrete. The test result shows that the average strength of gangue concrete is 40.7 MPa. It proves that the compressive strength of gangue concrete can exceed 40 MPa. The ordinary concrete also meets its expectant strength. The process from initial load to concrete rupture can be classified as compaction stage, linear elastic stage, non-linear elastic stage, microcracking stage, and instability failure stage.

**Figure 11:**
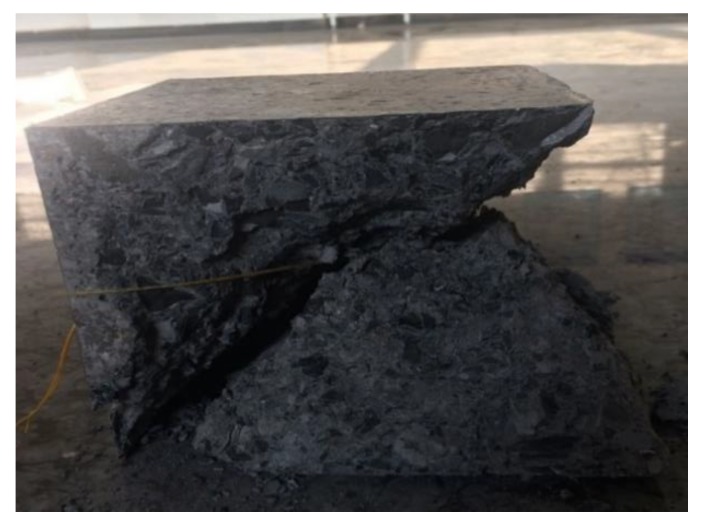
Fractured concrete.

(1) Compaction stage (OA). The intrinsic voids or cracks in concrete were compressed by the load. At this stage, the concrete’s macroscopic deformation is large, while the stress level is relatively low. The shape of the stress-strain curve is like a “concave”, which shows a non-elastic feature. (2) Linear elastic stage (AB). The intrinsic voids and cracks within the concrete have been compacted, and the structure that is comprised of mortar and aggregate bears most of the external loads, and the stress-strain curve is linear. Macro-cracks seldom appear at this stage. (3) Non-linear elastic stage (BC). Stress-strain curve changed to non-linear. This stage is still elastic, because no residual deformation occurs after unloading. However, there are few cracks that began to initiate and propagate at this stage. (4) Microcracking stage (CD). The elastic module obviously decreases at this stage, and bearing capacity of concrete weakens irreversibly. Some cracks in concrete propagate steadily and gradually coalesce with each other. (5) Unstable fracture stage (DE). The specimen fractures immediately, and the rapid stress drop occurs. It is due to the lower stiffness of the loading plate. The macro fractures are generated at this stage, and the dominating mode of failure is a shear failure, as shown in Figure 11.

**Figure 12:**
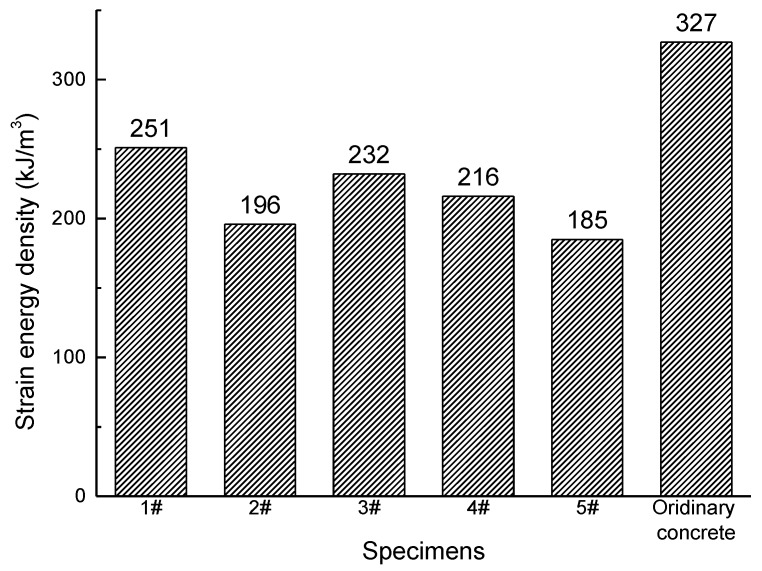
The strain energy density (before peak loading) of specimens.

The stress-strain curve of ordinary concrete is also shown in Figure 10. The strain energy density of five specimens before peak stress were calculated and shown in Figure 12. The strain energy density of ordinary concrete is obviously higher than the gangue concrete, which means the gangue concrete is much brittle than ordinary concrete.

### 4.2. Characteristic Parameters of AE

When concrete is damaged by external loads, it will go through the process of micro deformation, expansion, fractures transfixion, and instability failure. This process is very complicated, and it shows the weakening of concrete bearing capacity and the ultimate instability failure at the macro level. The strain energy generated by deformation can be released when microcracks initiate and propagate, and partial strain energy is released in the form of stress waves, which can be collected as AE signal. The strain energy changes with different damage stages of concrete, which form different AE signals. Thus, the damage levels of concrete can be evaluated by the AE monitoring method.

**Figure 13:**
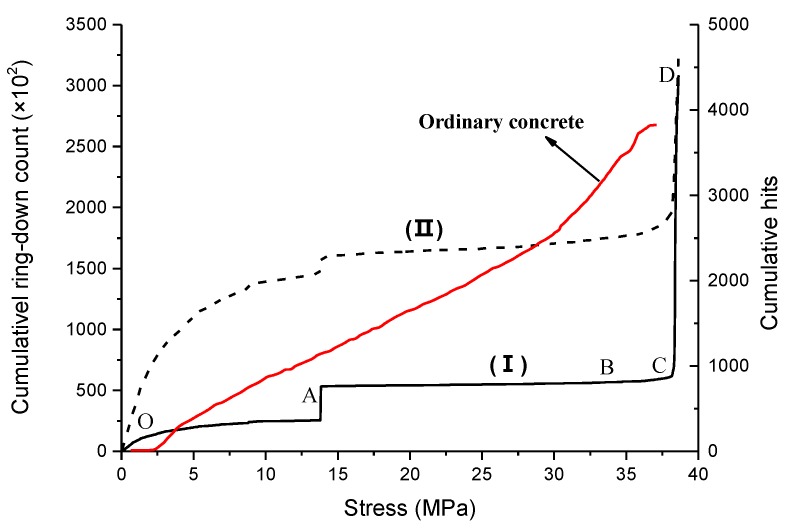
Cumulative ring-down count & cumulative hits-stress curve of concrete: (Ι) is cumulative ring-down count-stress curve, while (II) is cumulative hits-stress curve.

Among the characteristic parameters of AE, the hits and ring-down counts are often used to analyze the damage development process of the monitored specimen. A hit represents transmitting AE signals that exceed the threshold value and be counted by a channel. Thus, the cumulative hits reflect the quantity and frequency of the AE signal. Ring-down counts refer to the number of times that oscillating signals go over the threshold value, which can reflect the intensity of the AE signal. The highest signal-to-noise ratio AE signal was discussed in this paper. Cumulative ring-down counts & cumulative hits-stress curve of gangue concrete is shown as Figure 13, and the cumulative ring-down counts of ordinary concrete are also added in this figure for comparison. It is obvious that both cumulative hits and cumulative ring-down counts have a similar variation law during the test, which increases rapidly at points B and C. It indicates a large number of cracks initiating or developing at this time.

(1) Compaction stage (OA). At this stage, the AE accumulative ring-down counts and accumulative hits monotonously increase, while the growth rate keeps decreasing. It is because of the uneven and protruding surface of the specimen, as well as the damage at corners [30]. Besides, the deformation of the internal void and relative slide of aggregates can also generate the stress waves. As more and more voids are compacted, the magnitude of stress waves becomes smaller, which causes the slope of curve decreasing. (2) Linear elastic stage (AB). At the beginning of this stage, two parameters both increase rapidly as a result of the concentrated stress at the tip of preexisting voids, which leads to a small number of microcracks initiating. However, the stress is so small that these microcracks cannot be steadily developed. Thus, the AE signals are scarce and weak most of the time during this stage. (3) Microcracks initiating and developing stage (BC). The accumulative hits grow at a faster rate, while the accumulative ring-down count does not change significantly. This indicates that a large number of hits with short duration appear. Many microcracks appear and extend in the concrete specimen, and the crushed gangue aggregates may also be fractured at this stage. (4) Unstable fracture stage (CD). The macro fractures are formed, and the magnitude of stress waves increase rapidly, which cause the specimen’s failure in a short time.

The accumulative hits of ordinary concrete changes with the uniaxial compressive load are also given in Figure 13. It is obvious that there are some differences between gangue concrete and ordinary concrete. First, the ordinary concrete has a shorter compaction stage, and the AE signals are weaker. The reason for this phenomenon is that the fine aggregates in ordinary concrete have uniform shape and size, which cause its initial porosity to be much lower. Second, the accumulative hits of ordinary concrete are more active at the linear elastic stage, which represent more microcracks appearing and developing in the ordinary concrete at the linear elastic stage. The steady increases of accumulative hits at the elastic stage of concrete had also been observed in many previous researches [31,32,33], which is quite a difference with gangue concrete. These hits were generated by microcracks development or friction in mortar. The gangue concrete’s mortar contains small angular gangue particles, while the ordinary concrete’s mortar contains more spherical sand. The spherical sand can easily rotate in the mortar, which causes more microcracks and friction. Third, the growth rate of accumulative hits of ordinary concrete near the peak loading is obviously smaller than gangue concrete. It indicates that the damage development of gangue concrete damage is more abrupt.

**Figure 14:**
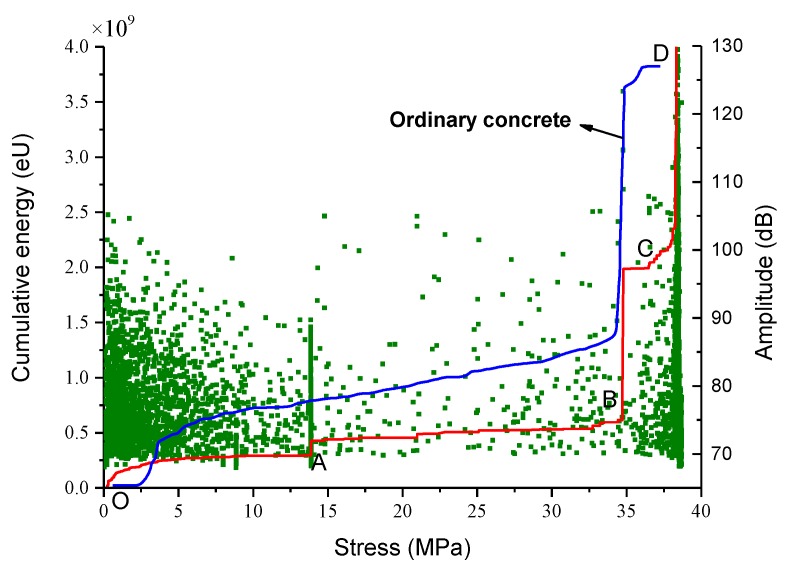
Amplitude & accumulative energy-stress curve of gangue concrete and ordinary concrete: the lines represent accumulative energy-stress curve, while the points represent amplitude.

The amplitude of the AE signals is the maximum voltage value in a hit, which can be used to represent the AE events intensity. Accumulative energy is the area under the envelope line of AE signals, which is a frequently-used parameter to measure the magnitude of AE event. Figure 14 is the amplitude-stress and accumulative energy-stress curve of gangue concrete and ordinary concrete. This curve can also be divided into four stages. It should be noted that the sharp increase at BC does not appear in the curve of Figure 14.

(1) OA section. The accumulative energy increases at first, and then gradually becomes stable. The hits have a large quantity and their amplitude varies greatly, which indicates the AE events at this stage are mainly caused by the uneven and protruding surface of the specimen, as well as the damage at the corners of the specimen. (2) AB section. A large number of hits with different amplitude appear near point A, which cause the accumulative energy to increase rapidly. These stress waves are released by the tip of pores that are compacted. After point A, the number of hits and the increment of accumulative energy is both smaller, which indicates the beginning of linear elastic. (3) BC section. The accumulative energy accelerated growth at this stage, and an abrupt increase appears near point B. It should be noted that the number of hits does not change significantly, and the abrupt increase is due to the appearance of some hits with a large amplitude. Macro fractures appear at this stage, and some gangue aggregates with large size ruptured, which release a lot of strain energy and cause a significant increase of accumulative energy. It should be noted that the stress of point C is 34.8 MPa, which is about 90.8% of peak stress. After point C, the number of hits meets significant growth, but their values are small, which causes the growth rate of the accumulative energy-stress curve to decrease. It indicates that, after the gangue aggregates rupturing, the macro fracture develops steadily. (4) CD section. The accumulative energy continues to increase at a higher rate. A large number of hits with variety amplitude magnitudes appear, which indicates there have both macro factures coalescence and microcracks appearing and developing. Finally, these fractures cause the failure of the gangue concrete specimen.

The accumulative energy of ordinary concrete changes with the uniaxial compressive load is also given in Figure 14. It is similar to the cumulative ring-down counts (Figure 13) that ordinary concrete specimens have shorter duration and weaker AE signals than gangue concrete at the linear elastic stage. It can be seen that there are two cumulative energy jumps (points B and D) before the final rupture of gangue concrete, while the ordinary concrete only has one. The cumulative energy jump at point B can be attributed to the rupture of gangue aggregates. This is due to the litholody, higher porosity, and microcracks of the coal gangue. Thus, micro-defects are more easily initiated and developed in crushed gangue concrete, which is opposite to what happened in ordinary concrete. As a result, a significant difference between ordinary concrete and gangue concrete is that the aggregates in gangue concrete may rupture during loading, which can be used to forecast damage of gangue concrete structure.

## 5. Conclusions

The gangue concrete, where coarse and fine aggregates are replaced by crushed gangue, was cast in this study. The uniaxial compressive tests along with AE monitoring were conducted, and the mechanical and AE characteristic parameters were obtained. The differences of AE characteristic parameters between ordinary concrete and gangue concrete were also analyzed. This paper obtained the following main results.

1. The mechanical properties of coal gangue are lower than gravel. This is due to the lithology, higher porosity, and microcracks of the coal gangue.
2. The mechanical test result shows that the maximum bearing strength of gangue concrete can reach 35–40 MPa.
3. Comparing with ordinary concrete, the AE signals of gangue concrete in the linear elastic stage is inactive. It is due to the mortar with angular gangue particles can not easily rotate, thus fewer microcracks appear and develop. The greater growth in AE signal strength is observed near the peak load as a result of the lower value of accumulative hits in the linear elastic stage.
4. In the Acoustic Emission (AE) test, the accumulative energy of gangue concrete has two jumps before the peak load, while ordinary concrete has one. The first jump of the accumulative energy of gangue concrete is caused by the rupture of gangue aggregates. Therefore, this difference can be used to forecast damage to the gangue concrete structure.

## Figures and Tables

**Table 1:** Grading parameters of gangue concrete.

Grading Type	W/C (%)	s/a (%)	Weight Per Unit Volume (kg/m^3^)			
			CG/G	S	C	W
Gangue concrete	45	25	1255	418	418	188
Ordinary concrete	45	42	1242	558	373	168

W/C—water-to-cement ratio, s/a—volume of sand aggregate to total aggregate, W—water, C—cement, S—sand, CG—coal gangue/gravel.

**Table 2:** Mineral Composition of Gangue (%): Q (Quartz)—SiO_2_, K (Kaolinite)—Al_4_(OH)_8_Si_4_O_10_, I (Illite)—KAl_2_(OH)_2_(AlSi)_4_O_10_, I/S—mixing layer of Illite & Smectite, S (Smectite)—(Na, Ca)_0.7_(Al, Mg)_4_(OH)_4_(SiAl)_8_O_20_·nH_2_O, Cl (Chlorite)—(Mg, Fe, Al)_6_(OH)_8_(Si, Al)_4_O_10_, F (Feldspar)—(Na, Ca) AlSi_3_O_8_/(Na, K) AlSi_3_O_8_, D (Dolomite)—(Ca, Mg)CO_3_, C (Calcite)—CaCO_3_, L (Siderite)—FeCO_3_, P (Pyrite)—FeS_2_.

Mineral Composition	Q	K	I	I/S	S	Cl	F	D	C	L	P	Coal
Gangue	20	33	10	11	2	2	0.5	0.5	0.5	1.3	2.6	15

**Table 3:** Chemical component of gangue.

Chemical Component	Na_2_O	MgO	Al_2_O_3_	SiO_2_	K_2_O	CaO	Fe_2_O_3_	P	S
Gangue	0.41	1.4	20.7	53.7	2.0	1.3	6.7	0.05	1.53
Chemical component	F	Ba	Mn	Cu	Pb	Zn	Ti	Cl	
Gangue	<0.045	≤0.002	0.043	0.0005	<0.0002	0.005	0.42	0.009	
